# Supplementary material for: The Impact of COVID-19 on Gastrointestinal Motility Testing in Asia and Europe
Source: J Clin Med. 2020 Oct 1;9(10):3189. doi: 10.3390/jcm9103189 (PMC7600249; doi:10.3390/jcm9103189)
Supplement: Supplementary file 1 [file jcm-09-03189-s001.pdf]

Table S1. Total cases and deaths in each country.

	Total Cases *	Total Deaths *	Total Cases Per 100,000*	Mortality Rates Per 100,000 *	Covid-19 Government Response Stringency Index #
**Asian countries**					
China	83,075	4634	58	3	78.24
Hong Kong	1109	4	148	0.5	52.78
Japan	17,332	922	137	7	26.85
Korea	12,051	277	235	5	56.94
Taiwan	443	7	19	0.3	22.22
Thailand	3134	58	45	0.8	62.96
Philippines	25,392	1074	232	10	77.78
Malaysia	8445	120	261	4	57.41
Singapore	40,197	25	6874	4	77.78
**European countries**					
Belgium	59,918	9650	5171	833	78.70
Croatia	2249	107	547	28	89.81
Denmark	12,099	594	2225	105	65.74
France	156,287	29,374	2395	450	87.96
Germany	187,251	8863	2235	106	64.35
Ireland	25,250	1705	5170	353	90.74
Israel	18,795	300	2004	34	77.78
Italy	237,828	34,448	3933	570	62.96
Norway	8620	242	1589	33	64.81
Poland	28,577	1222	755	32	83.33
Portugal	36,180	1505	3548	147	75.00
Romania	21,404	1380	1112	72	87.04
Russia	511,423	6715	3516	46	85.19
Spain	290,289	28,275	6392	607	81.94
Sweden	51,171	5045	5053	499	46.30
Switzerland	31,063	1938	3589	227	69.44
The Netherlands	48,461	6053	2829	358	79.63
Turkey	175,218	4778	2083	51	75.93
United Kingdom	292,950	41,481	4316	611	79.63

* Cited 12 June 2020 (https://www.worldometers.info/coronavirus/); # Cited 12 June 2020 (https://www.bsg.ox.ac.uk/research/research-projects/oxford-covid-19-government-response-tracker).
